# Differentially expressed microRNAs in the serum of cervical squamous cell carcinoma patients before and after surgery

**DOI:** 10.1186/1756-8722-7-6

**Published:** 2014-01-10

**Authors:** Wen-Tao Wang, Ya-Nan Zhao, Jin-Xing Yan, Mei-Ying Weng, Yan Wang, Yue-Qin Chen, Shun-Jia Hong

**Affiliations:** 1 Key Laboratory of Gene Engineering of the Ministry of Education, State Key Laboratory for Biocontrol, Sun Yat-sen University, Guangzhou 510275, China; 2 Guangdong Provincial Key Laboratory of Malignant Tumor Epigenetics and Gene Regulation, Department of Obstetrics & Gynecology, Sun Yat-sen Memorial Hospital, Sun Yat-sen University, Guangzhou 510120, P. R. China; 3 Department of Obstetrics & Gynecology, Hainan General Hospital, Haikou 570012, China

**Keywords:** Circulating microRNA, Cervical SCC, Serum, Tumor biomarkers, Post-therapeutic monitoring

## Abstract

**Background:**

The purpose of this study was to detect the serum microRNAs (miRNAs) that are differentially expressed in cervical squamous cell carcinoma (SCC) patients and negative controls, with a focus on the miRNA profiles of the patients before and after surgery. The aim of the study is to evaluate the potential of these miRNAs as novel markers for the post-therapeutic monitoring of cervical SCC patients.

**Results:**

A total of 765 serum miRNAs from 10 cervical SCC patients before surgery, 10 cervical SCC patients after surgery, and 10 negative controls were profiled using a TaqMan MicroRNA Array. A set of selected differentially expressed miRNAs were further analyzed in the patients at different perioperative periods, including preoperative, 1 week postoperative, and one month postoperative. The results showed that several serum miRNAs were differentially expressed in the cervical SCC patients compared with the negative controls, including miR-646, miR-141* and miR-542-3p. More importantly, we found that levels of specific serum miRNAs were deregulated in the pre- and postoperative stages, and these miRNAs could be useful for post-therapeutic monitoring of disease progression. Finally, we depicted a regulatory network of differentially expressed serum miRNAs, and many possible target genes were predicted in the estrogen-mediated signal pathways, supporting the hypothesis that cervical SCC is a hormone-associated gynecological disease.

**Conclusions:**

Our study demonstrated that the circulating miRNAs miR-646, miR-141* and miR-542-3p could potentially serve as non-invasive biomarkers for cervical SCC. The levels of these specific miRNAs might be useful for the post-therapeutic monitoring of disease progression. This is the first report showing that circulating miRNAs could serve as biomarkers for the therapeutic intervention of cervical SCC.

## Introduction

Cervical squamous cell carcinoma (cervical SCC) is a common cervical cancer type that is closely correlated with high-risk human papillomavirus (HPV) infection [1], [2]. Cervical SCC is the third most malignant carcinoma that affects women's health and is the leading cause of death from cancer in women worldwide. Due to standardized screening of cervical cancer, the morbidity and mortality rates of cervical SCC have decreased [3]. However, due to patients’ resistance to therapeutic interventions, the annual number of cervical cancer deaths has continued to rise and is more prevalent in developing countries [4]. Therefore, an increased understanding of the molecular mechanisms and the screening of predictive biomarkers for cervical SCC progression and metastasis is necessary.

MicroRNAs (miRNAs) are a class of small non-coding RNAs that are approximately 22 nucleotides in length and regulate gene expression post-transcriptionally through translational repression or transcript cleavage [5], [6]. Many studies have shown that miRNAs are related to cell processes [5], [6], and the dysregulation of these events can lead to the occurrence of a number of diseases, such as lung cancer [7], prostate cancer [8], colorectal tumor [9], leukemia [10], and cervical cancer [11]. The miRNA research in the past 20 years, including the development of sensitive molecular detection techniques in the last 5 years, has allowed researchers to investigate miRNAs in circulation and in body fluids, such as serum [12], human whole saliva [13], and urine [14], initiating a new era of disease research.

A pilot study by Roth et al. demonstrated that the concentration of miR-155 in sera can be a significant index to distinguish patients with primary breast cancer from healthy women, and the levels of circulating miR-34 could indicate the disease progression stage [15]. A growing number of investigations of circulating miRNAs have revealed the remarkable potential value of circulating miRNAs for the diagnosis and prognosis of diseases, especially cancer [16-19]. Our previous study also showed that circulating miR-122, miR-145*, miR-199a and miR-542-3p could potentially act as noninvasive biomarkers for endometriosis [20]. Although circulating miRNAs have been described as important non-invasive biomarkers with high sensitivity and specificity in many other diseases, the study of circulating miRNA in cervical cancer is limited [21], [22], and no studies have reported miRNA expression patterns in cervical cancer patients before and after surgery.

In this study, we screened the differentially expressed miRNAs in the serum of cervical cancer patients, with a focus on the miRNA profiling before and after surgery. The aim of the study is to screen for the predictive biomarkers that could be useful for post-therapeutic monitoring of cervical SCC patients.

## Material and Methods

### Patient and sample material

Serum samples and clinicopathologic data were collected from 10 primary cervical SCC patients who underwent radical hysterectomies with pelvic lymph node dissections in 2008 in the Department of Gynecology at Hainan Provincial People’s Hospital (Haikou, China). The mean age was 51.0 years (range, 34-78 years). Of these 10 patients, no patients received chemotherapy or radiotherapy prior to surgery. Blood samples were collected before surgery, and then one week and one month after surgery. According to the 5-year follow-up, 5 of the 10 patients were still alive, but the other patients had poor prognoses.

The clinicopathologic data are shown in Table 1. The control serum samples were collected from 15 volunteers who sought diagnostic laparoscopy due to infertility in the Department of Gynecology, Sun Yat-sen Memorial Hospital (Guangzhou, China) in 2011. All volunteers were diagnosed with fallopian tube disease with neither cervical intraepithelial neoplasia nor HPV infection. The mean age of the volunteers was 28.8 years (range 24-34 years). None of the 15 women were treated with hormone therapy in the 3 months before the operation, and serum samples were taken 1-3 days prior to the laparoscopic procedure. All patients provided informed consent, and the study was approved by the ethics committees of Sun Yat-sen University and Hainan Provincial People’s Hospital.

**Table 1:** Clinicopathologic Characteristics of CSCC* patients

	**Favorable prognosis**	**Poor prognosis**
**Patient age**		
**≤35**	**1**	**0**
**>35**	**4**	**5**
FIGO** stage		
**Ib1**	**5**	**4**
**IIa**	**0**	**1**
**Differentiation**		
**Well**	**0**	**1**
**Moderate**	**0**	**1**
**Poor**	**5**	**3**
LNM***		
**Yes**	**0**	**0**
**No**	**5**	**5**
**Stromal invasion**		
**≤1/2**	**5**	**1**
**>1/2**	**0**	**4**
**Vaginal wall extension**		
**Yes**	**0**	**2**
**No**	**5**	**3**
**Parametrial extension**		
**Yes**	**0**	**0**
**No**	**5**	**5**
LVSI****		
**Yes**	**0**	**1**
**No**	**5**	**4**

*CSCC - Cervical Squamous Cell Carcinoma.

**FIGO - International Federation of Gynecology and Obsterics.

***LNM - Lymph Node Metastasis.

****LVSI - Lymph Vascular Space Invasion.

### Serum harvested and RNA extraction

Fasting blood samples were collected from all donors and were separated into serum and cellular components within 1 h at 3000 rpm at 4°C for 10 minutes after being left to clot at room temperature. The sera were stored at -80°C until RNA extraction. Isolation of total RNA from 1 ml of serum was conducted according to the manufacturer’s instructions of the mirVana PARIS Kit (Ambion, Austin, TX). The RNA samples were then frozen at -80°C.

### Quantitative real-time PCR

Using the Taq-Man miRNA array, we initially profiled 768 miRNAs from two pooled samples, including a pool of 10 cervical SCC samples and a pool of 15 negative controls. To quantify the accurate amounts of miRNAs in the serum of samples, we reverse transcribed 50 ng total RNA for each sample using the ReverTra Ace qPCR RT Kit (Toyobo, Osaka, Japan). The miRNA levels were determined in triplicate using SYBR Premix Ex Taq II-based (Takara, Kyoto, Japan) quantitative real-time PCR with Roche Light Cycler 480II (Roche, Switzerland). U6 small nuclear RNA, a common internal control used in studies quantifying circulating miRNAs, was used to normalize the relative expression of miRNAs [23], [24].

### Statistical analysis

GraphPad Prism software (version 5.0) was used to perform the statistical analyses and generate figures. The heat map was analyzed using Cluster 3.0 and TreeView software. Cytoscape (version 3.0.1) was used to network the miRNAs and the target genes. The final figures were generated using Adobe Illustrator CS5. We determined the significance of the differentially expressed miRNA levels between two groups (patient and negative control) using Fisher’s exact test and the Mann–Whitney *U*-test, while the related samples (pre- and postoperative) were analyzed using Wilcoxon signed rank test and three groups and multiple comparisons using the Kruskal-Wallis test and the least significant difference test after ranking the relative expression, respectively. In this study, the P < 0.05 with two tails was considered statistically significant.

## Results

### A significant number of miRNAs are present in serums of patients with cervical SCC

To identify the potential clinical applications of circulating miRNAs, TaqMan arrays were used to screen miRNAs from two pools of serum samples including 10 cervical SCC patients and 10 healthy women. The results showed that the levels of 291 of the 338 detectible circulating miRNAs were more than two-fold change in the cervical SCC serum samples than the negative control serums (Additional file 1: Table S1). We identified 40 upregulated and 26 downregulated miRNAs that showed differential expression profiles greater than ten-fold in the pooled cervical SCC serum sample compared with the negative control (Additional file 2: Table S2). These miRNAs include miR-21-5p [25], miR-143-3p [25] and miR-214-3p [26], which has been reported as being related to cervical SCC. MiR-646 was the most abundant miRNA species in the cervical SCC serum sample, while miR-508 was the most downregulated miRNA.

**Figure 1:**
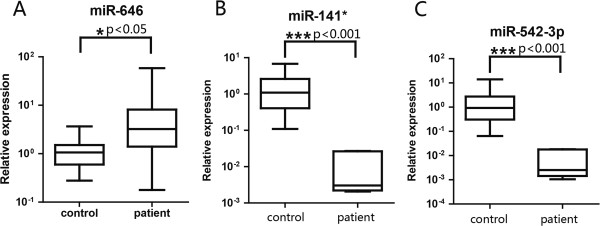
**Differentially expressed miRNAs in the serum of cervical SCC patients and control samples.** The miRNA expression levels in serum from cervical SCC patients (n = 10) and negative controls (n = 15). The expression profiles of miR-646 **(A)** were significantly higher in cervical SCC patients (P < 0.05), whereas those of miR-141* **(B)**, and miR-542-3p **(C)** were lower in cervical SCC patients (P < 0.001). The indicated P values were determined by a two-tailed Mann–Whitney *U*-test, * p < 0.05, *** p < 0.001.

To confirm the accuracy of the genome-wide screening, 5 of the most dysregulated miRNAs were selected for a validation sample set (10 for cervical SCC and 15 for negative control). Among the selected miRNAs, 3 of the 5 were significantly different in the cervical SCC serum samples when compared to the control group: miR-646 (upregulated, p < 0.05, Figure 1A), miR-141* (downregulated, p < 0.001, Figure 1B) and miR-542-3p (down-regulated, p < 0.01, Figure 1C). These results suggested that specific serum miRNAs could serve as informative biomarkers for the cervical SCC, though a larger cohort of samples is required to validate this observation.

### Differentially expressed miRNAs in the serum of cervical SCC patients before and after surgery

We next asked if the miRNAs were different in the serum of the patients before and after surgery. To evaluate the expression of serum miRNAs in the perioperative periods, we further profiled miRNA spectra from pools of 10 cervical SCC serum samples before surgery and 10 serum samples after surgery. We identified 765 miRNAs during this analysis process. In this profile, the expression levels of 133 miRNAs were found to be increased more than two-fold in the serum 1 week after surgery compared to that before operation, while 81 microRNAs were decreased after surgery (Additional file 3: Table S3).

**Figure 2:**
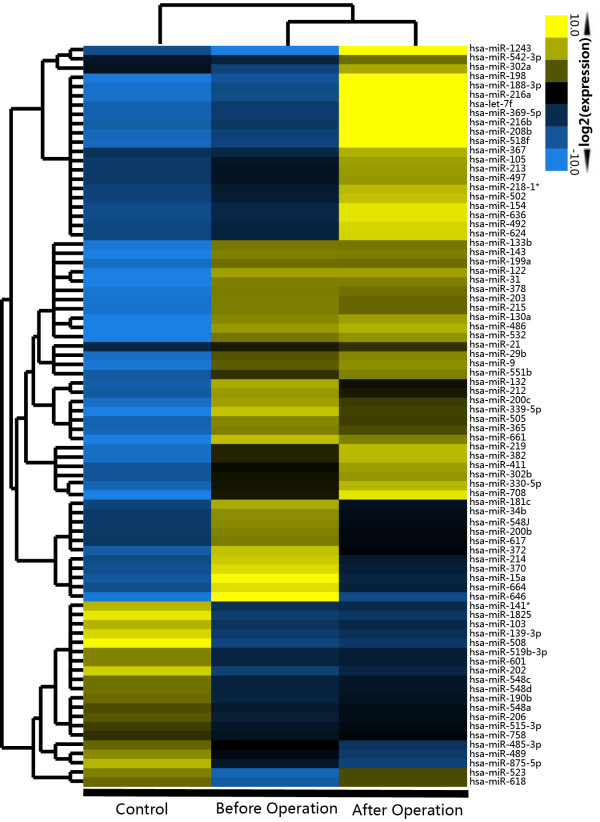
**The heat map displays the top 80 microRNAs with the most differential expression levels in the control and the patients before and after surgery.** The average level for each of the 3 miRNA serum samples was set as 1. The fold changes of individual miRNAs of the control and before or after surgery against the average miRNA level were analyzed by unsupervised hierarchical clustering.

**Figure 3:**
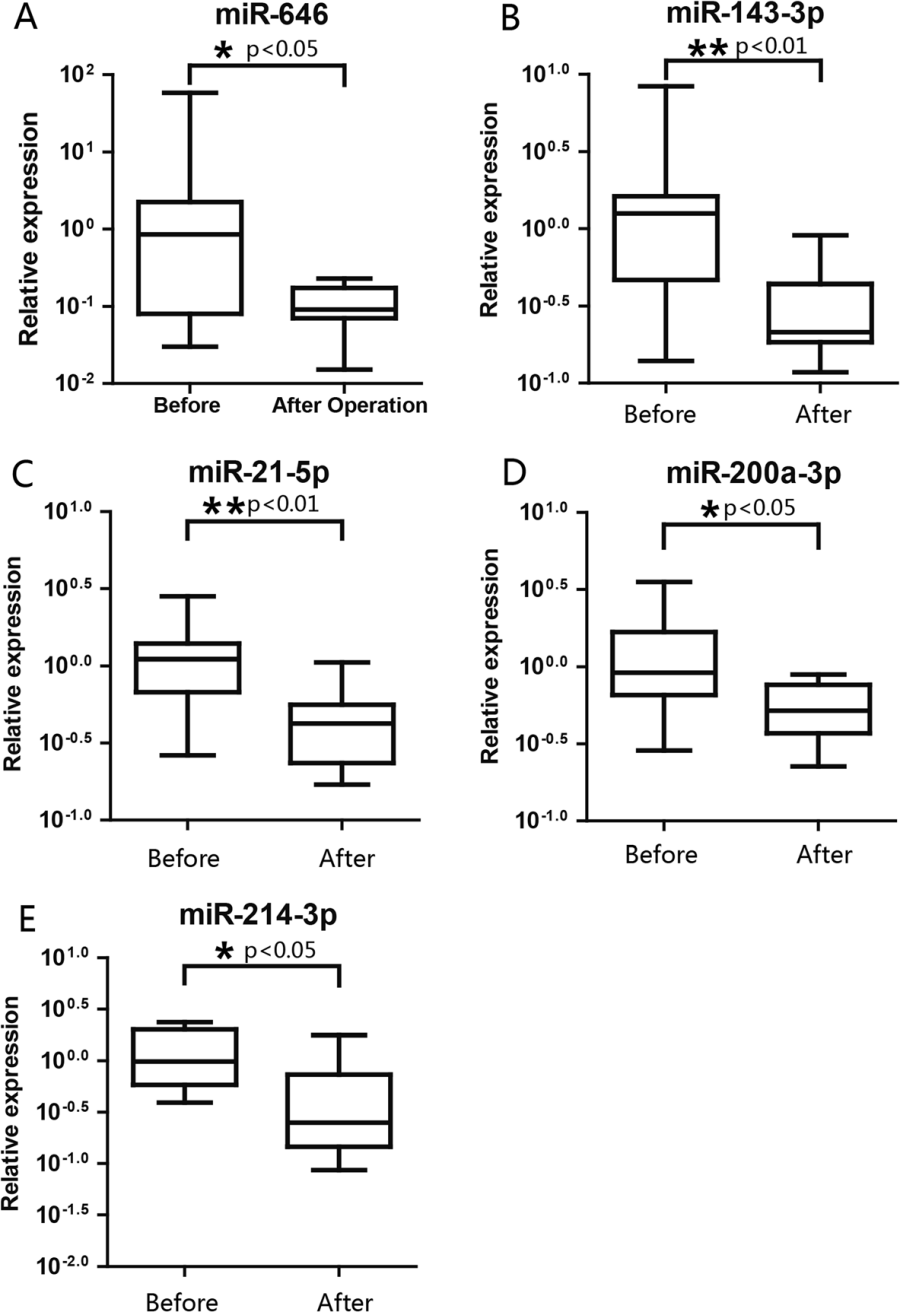
**The differential expressed miRNAs in the matched serum samples from cervical SCC patients before and after surgery.** MiR-646 **(A)** is expressed a higher level in the serum of cervical SCC patients after surgery than that before surgery, whereas, miR-143-3p **(B)**, miR-21-5p **(C)**, miR-200a-3p **(D)**, and miR-214-3p **(E)** are expressed a lower level postoperatively.

Figure 2 illustrates the most differentially expressed miRNAs in the whole serum sample among the negative control and the cervical SCC patient before and after surgery. Unsupervised hierarchical clustering was used to separate the samples and miRNAs into different groups. Additional file 4: Table S4 exhibited a greater than ten-fold change between the cervical SCC pooled serum samples before and after surgery, including the most abundant miRNA, miR-1243, and the most downregulated miRNA, miR-646. In the validation test, 9 differentially expressed miRNAs were chosen for further study with the 10 matched-pair serum samples before and after surgery. Among the selected miRNAs, 5 of the 9 could clearly distinguish cervical SCC serum samples after surgery with high confidence (p < 0.05): miR-646 (Figure 3A), miR-143-3p (Figure 3B), miR-21-5p (Figure 3C), miR-200a-3p (Figure 3D) and miR-214-3p (Figure 3E). Differentially expressed miRNAs in the serum of cervical SCC patients before and after surgery suggest that the expression levels of serum miRNAs have potential to serve as novel biomarkers for monitoring the therapy outcome during the perioperative period for cervical SCC.

### Levels of specific miRNAs might be used for post-therapeutic monitoring of the cancer in progression

The observation above indicated that the serum miRNAs were differentially expressed in patients before and after surgery. To further investigate if levels of specific circulating miRNAs could be used to monitor the therapy outcome or post-therapeutic progression, eight miRNAs (miR-132, miR-181c, miR-15a, miR-370, miR-143-3p, miR-21-5p, miR-200a-3p, and miR-646) were used to perform the subsequent qRT-PCR validation in the different perioperative periods of the same patients, including preoperative, 1 week postoperative, and one month postoperative. These miRNAs were selected based on the highest difference in expression or whether they had been previously reported to function in cervical SCC.

**Figure 4:**
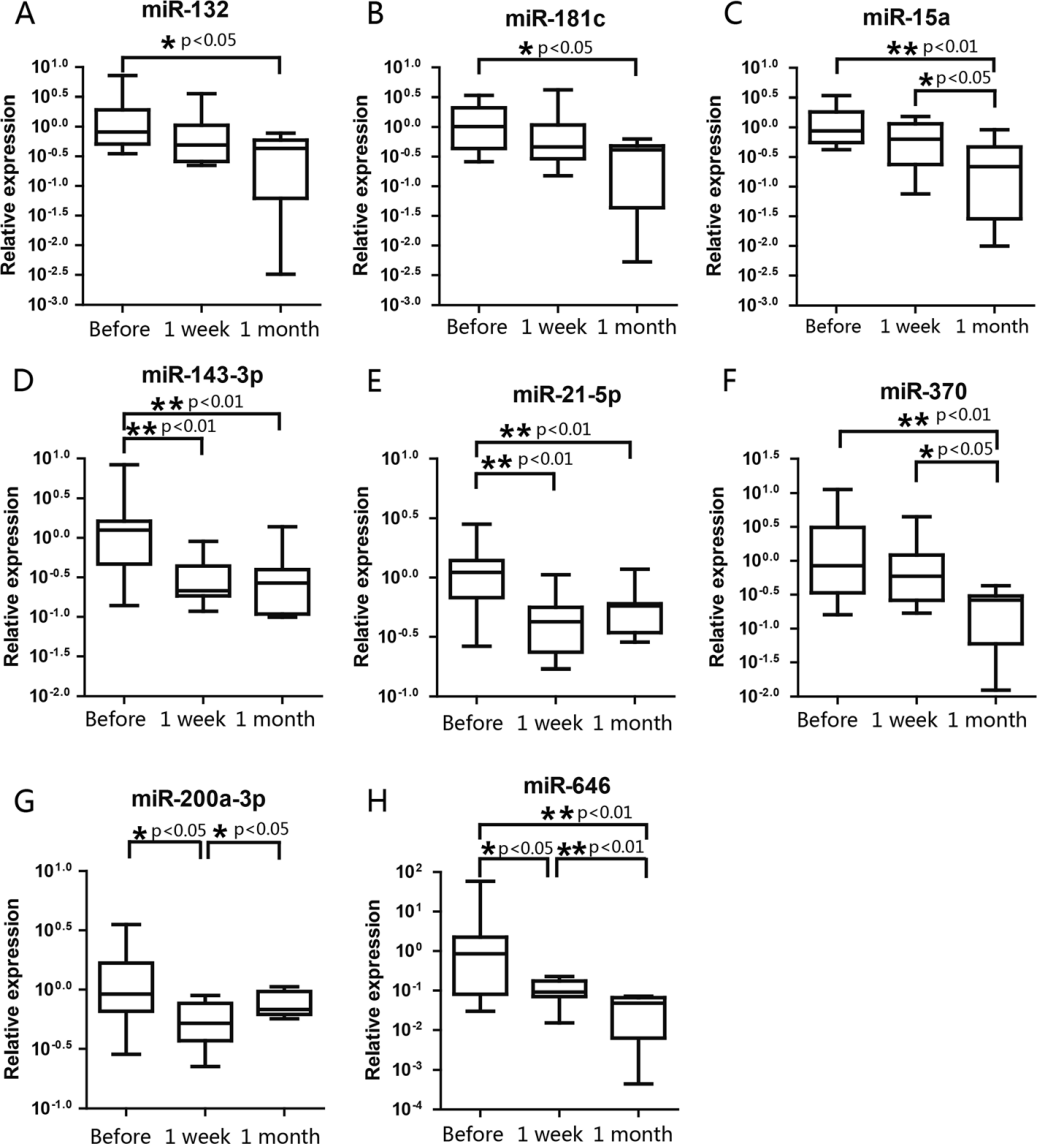
**The expression levels of specific circulating miRNAs were significantly different between the serum samples at the preoperative, 1 week or one month after surgery.** MiR-132 **(A)** and miR-181c **(B)** was decreased significantly one month after surgery compared to the preoperative level; no significant difference between the other two groups. The expression levels of miR-15a **(C)** and miR-370 **(F)** were significantly decreased 1 month after surgery compared to the preoperative levels and 1-week postoperative levels; no significant differences were observed between the preoperative and one week postoperative levels. The levels of miR-143-3p **(D)** and miR-21-5p **(E)** were significantly decreased 1 week and 1 month after surgery compared with the preoperative levels; no significant differences between the of one week postoperative and one month postoperative levels were. The expression levels of miR-200a-3p **(G)** was decreased significantly 1 week after surgery compared to the preoperative levels, but increased significantly when compared to 1 month postoperative levels. The expression levels of miR-646 **(H)** were significantly different between all three time points.

Four miRNAs (miR-132 (p < 0.05), Figure 4A; miR-181c (p < 0.05), Figure 4B; miR-15a (p < 0.01), Figure 4C; and miR-370 (p < 0.01), Figure 4F) were found to significantly decrease one month after surgery compared to the preoperative levels. Other three miRNAs (miR-143-3p, Figure 4D; miR-21-5p, Figure 4E; and miR-200a-3p, Figure 4G) showed significant differential expression levels between preoperative and postoperative patients (p < 0.01). Notably, among the cervical SCC patient serum samples, the relative concentration of miR-646 (Figure 4H) was found to steadily decrease 1 week and one month after surgery compared to the preoperative samples. These results suggest that the levels of specific circulating miRNAs could be used for monitoring the therapy outcome or post-therapeutic monitoring of cervical SCC in progression. Further studies with larger cohort of clinical samples, especially different therapeutic period samples are necessary.

### Regulation network of differentially expressed serum miRNAs in the enriched pathways and processes of cervical SCC

**Figure 5:**
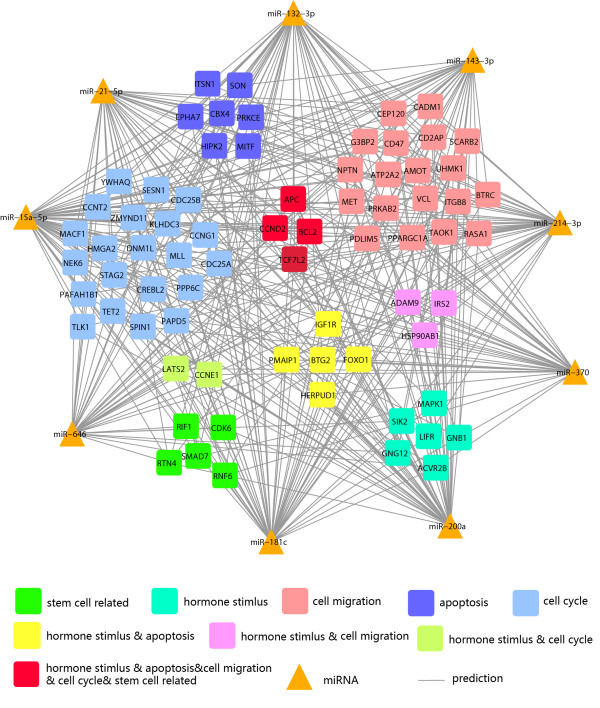
**Network of microRNAs and their target genes associated with the biological pathway and the diagnosis of cervical SCC.** (Node Shape: triangle for microRNA, square for target gene; edges for relationship.)

The variation in serum miRNA expression levels before and after surgery suggested an association between miRNAs and the key proteins underlying mechanisms related to the therapy. To better understand the possible biological pathways that the serum miRNAs might be involved in, we constructed a miRNA-mRNA network to search the miRNA target genes that might function in the cervical SCC process. Analysis using the StarBase database [27], the Database for Annotation, Visualization and Integrated Discovery (DAVID) [28], and a Fisher exact test uncovered the enriched biological pathways, including cell cycle and proliferation (p < 0.001, 57 genes); hormone stimulus response (p < 0.001, 18 genes); cell migration regulation (p < 0.02, 31 genes); apoptosis (p < 0.03, 20 genes); and stem cell related pathways (p < 0.05, 8 genes). A global network of the selected miRNAs and their target genes (or potential target mRNAs) is depicted in Figure 5. All of genes possess more than 5 edges, and many possible target genes were predicted in the estrogen-mediated signal pathways. For example, insulin-like growth factor 1 (IGF1R) shares important signaling cascades with estrogen receptor [29], while forkhead box O1 (FOXO1) is involved in the estrogen mediated E2/PI3K/Akt/FOXO1 pathway [30]. It has been reported that cervical SCC is a hormone-associated gynecological disease [31].

Notably, a number of miRNAs were related to chemotherapy drug responses or regulation of cell adhesion and migration (Figure 5, red and purple box indicated). For instance, miR-143-3p, miR-181c-5p, miR-200a-3p, miR-21-5p, and miR-370-3p were predicted to target metallopeptidase domain 9 (ADAM9). ADAM9 has been reported to be involved in the biological pathway of chemotherapy [32] and involved in the biological processes such as cell adhesion and migration in the cervical SCC [33]. Another example is BCL2, which contains the reverse complements to miR-15a-5p and miR-181c in the 3’UTR, responds to anthracycline combination (ATC) chemotherapy [34], and plays an important role in cervical cancer progression [35]. These results showed a probable link between circulating miRNAs and estrogen-associated with the cervical SCC as well as chemotherapy.

## Discussion

Finding a non-traumatic biomarker for predicting the prognosis of cancer and monitoring the outcome of therapy has a significant importance to cancer research. Several factors have been reported to be predictive biomarkers for monitoring of cervical cancer recurrence, such as squamous cell carcinoma antigen (SCCA) [36], carbohydrate antigen 125 (CA125) [37], vascular endothelial growth factor (VEGF) [38] among others, but these markers have low sensitivity and specificity. HPV testing was also reported as a well accepted maker for IVD of cervical SCC worldwide. However, the recent progress has proposed that the efficiency of HPV DNA/RNA testing is dependent largely on the sampling method and cervical cancer screening using cytologic testing alone or together with HPV DNA/RNA testing is less-frequent [39]. Therefore, developing novel biomarkers for clinical diagnosis and therapeutic intervention require further research. While miRNA testing is based on serum, which is more stable and have less influence during sampling. In this study, we screen the differentially expressed miRNAs in the serum of cervical SCC patients, with a focus on the miRNA profiling before and after surgery. Notably, a number of serum miRNAs differentially expressed in the cervical SCC compared with the negative control, including miR-646, miR-141* and miR-542-3p. More importantly, we revealed that the levels of specific serum miRNAs that were differentially expressed in the patients before and after surgery; however, these results need to be confirmed with larger samples. This study is the first report on circulation miRNAs in patients before and after surgery. Finally, we also depicted the regulation network of differentially expressed serum miRNAs and discussed the possible functions in which these miRNAs might be involved, which could help us to understand the molecular mechanisms underlying the high tumorigenicity of the cancer and to identify new therapeutic strategies.

In recent years, a number of studies have indicated that the presence of altered miRNA profiles in the serum for several cancer types and other diseases (15-20), suggesting that circulating miRNAs are potential novel biomarkers. Several studies have also shown that miRNAs may be useful in clinical management during the perioperative period. For instance, Ng et al. [40] found that the expression levels of serum miR-17-3p and miR-92 decrease postoperatively compared with the preoperative period in colorectal cancer patients. Similar results have been discovered in breast cancer patients [17]; the preoperative expression levels of miR-195 were significantly higher than those of healthy controls but decreased 2 weeks after surgery. Peng Qi et al. [41] showed that the expression level of miR-122 continues to decrease in hepatocellular carcinoma with chronic hepatitis B viral infection compared with preoperative and healthy people. MiR-218 was reported to be associated with cervical adenocarcinoma and lymphatic node metastasis and was reduced in the plasma compared with healthy people [21]. Together with the results of this study, it could be suggested that circulating miRNAs might be useful biomarkers for monitoring the therapy outcome or post-therapeutic monitoring of disease progression.

Cervical SCC is one of the most malignant carcinomas affecting women's health and leading death worldwide, and understanding the molecular mechanisms underlying the high tumorigenicity of this cancer is necessary. Previous research has demonstrated that some specific miRNAs contribute to the pathogenesis and process of cervical SCC. For example, Martinez et al. [11] illustrated that miR-218 is reduced by HPV type 16 (HPV-16) infection and mediates the expression level of LAMB3, which has provided a better understanding of the molecular mechanisms involved in cervical carcinogenesis.

In our work, a miRNA-mRNA predictive targeting network has been developed to search for the critical genes that function in the biological processes of cervical SCC. From a large amount of genes in the network, we found a total of 72 genes in mainstream biological pathways that are commonly targeted by 5 or more specific miRNAs. Interestingly, the selected miRNA target genes were predicted to affect main biological processes such as hormone-mediated signal pathways and chemotherapy responses. For example, BCL2, which responds to anthracycline combination (ATC) chemotherapy, is the target of miR-15a-5p and miR-181c and was found in the cervical SCC regulatory network. The results suggest that the miRNAs and their target genes might not only serve as novel diagnostic biomarkers but also as new therapeutic targets.

In conclusion, our study demonstrated that the circulating miRNAs could potentially serve as non-invasive biomarkers for cervical SCC. MiR-646, miR-141* and miR-542-3p levels were significantly different between cervical SCC serum samples and the control group. More importantly, the expression levels of miR-21, miR-200a, miR-143, miR-15a, miR-181c, miR-646, miR-132 and miR-370 in the serum can, to some extent, be used as biomarkers to monitor therapeutic efficacy. Further investigation with a larger cohort of samples is required to validate these observations. This study is the first report that circulating miRNAs could serve as biomarkers for the therapeutic intervention of cervical SCC.

## Abbreviations

miRNA: microRNA; SCC: Squamous cell carcinoma; HPV: Human papillomavirus; PCR: Polymerse chin rection; RT-PCR: Reverse transcription-PCR; P: Probability; DAVID: Database for Annotation, Visualization and Integrated Discovery; IGF1R: Insulin-like growth factor 1; FOXO1: Orkhead box O1; ADAM9: Metallopeptidase domain 9; ATC: Anthracycline combination; SCCA: Squamous cell carcinoma antigen; CA125: Carbohydrate antigen 125; VEGF: Vascular endothelial growth factor.

## Competing interests

The authors have no conflicts of interests.

## Authors' contributions

WTW and YAZ participated in research design and conducted experiments; JXY and MYW performed data analysis; YW conducted clinical data analysis; SJH and YQC participated in research design and drafted the manuscript. All authors read and approved the final manuscript.

## Supplementary Material

Additional file 1: Table S1 The global miRNA species showed relative expression profiles between cervical squamous cell carcinoma serum samples and negative controls. Click here for file

Additional file 2: Table S2 MiRNAs fluctuated more than ten folds between cervical squamous cell carcinoma serum samples and negative controls. Click here for file

Additional file 3: Table S3 The global miRNA species showed relative expression profiles between cervical squamous cell carcinoma serum samples before and after surgery. Click here for file

Additional file 4: Table S4 MiRNAs fluctuated more than ten folds between cervical squamous cell carcinoma serum samples before and after surgery. Click here for file
