# Supplementary material for: Differentially expressed microRNAs in the serum of cervical squamous cell carcinoma patients before and after surgery
Source: J Hematol Oncol. 2014 Jan 10;7:6. doi: 10.1186/1756-8722-7-6 (PMC3892020; doi:10.1186/1756-8722-7-6)
Supplement: Additional file 1: Table S1 — The global miRNA species showed relative expression profiles between cervical squamous cell carcinoma serum samples and negative controls. [file 1756-8722-7-6-S1.docx]

**Table S1.** **The global miRNA species showed relative expression profiles**

**between cervical squamous cell carcinoma serum samples and negative**

**controls**

| Global Downregulated microRNA | Fold Change | Global Upregulated  microRNA | Fold Change |
| --- | --- | --- | --- |
| hsa-miR-508 | 3.894E+04 | **hsa-miR-646** | 3.04E+07 |
| hsa-miR-1825 | 1.606E+04 | **hsa-miR-1183** | 1.83E+07 |
| hsa-miR-202 | 1.061E+04 | **hsa-miR-1233** | 1.15E+06 |
| hsa-miR-139-3p | 9.467E+03 | **hsa-miR-661** | 1.08E+06 |
| hsa-miR-523 | 8.487E+03 | **hsa-miR-486** | 5.24E+05 |
| hsa-miR-618 | 2.307E+03 | **hsa-let-7a** | 5.02E+05 |
| hsa-miR-103 | 2.141E+03 | **hsa-miR-122** | 5.02E+05 |
| hsa-miR-141* | 2.023E+03 | **hsa-miR-663B** | 2.96E+05 |
| hsa-miR-875-5p | 5.322E+02 | **hsa-miR-339-5p** | 2.68E+05 |
| hsa-miR-1243 | 2.556E+02 | **hsa-miR-660** | 1.29E+05 |
| hsa-miR-335* | 2.461E+02 | **hsa-miR-130a** | 1.28E+05 |
| hsa-miR-601 | 2.434E+02 | **hsa-miR-15a** | 1.09E+05 |
| hsa-miR-519b-3p | 2.428E+02 | **hsa-miR-30a-3p** | 6.91E+04 |
| hsa-miR-548d | 1.416E+02 | **hsa-miR-942** | 3.60E+04 |
| hsa-miR-548c | 1.341E+02 | **hsa-miR-1303** | 3.53E+04 |
| hsa-miR-190b | 1.222E+02 | **hsa-miR-143** | 3.27E+04 |
| hsa-miR-489 | 72.113 | **hsa-miR-31** | 3.16E+04 |
| hsa-miR-206 | 61.472 | **hsa-miR-532** | 3.15E+04 |
| hsa-miR-548a | 33.213 | **hsa-miR-664** | 3.15E+04 |
| hsa-miR-191* | 30.756 | **hsa-miR-372** | 3.11E+04 |
| hsa-miR-515-3p | 17.646 | **hsa-miR-370** | 3.08E+04 |
| hsa-miR-195 | 16.435 | **hsa-miR-652** | 3.03E+04 |
| hsa-miR-302a | 16.270 | **hsa-miR-200c** | 3.01E+04 |
| hsa-miR-485-3p | 15.448 | **hsa-miR-425*** | 1.74E+04 |
| hsa-miR-192* | 15.408 | **hsa-miR-378** | 1.74E+04 |
| hsa-miR-758 | 9.898 | **hsa-miR-22*** | 1.72E+04 |
| hsa-miR-19a | 8.533 | **hsa-miR-133b** | 1.70E+04 |
| hsa-miR-190 | 8.242 | **hsa-miR-132** | 1.64E+04 |
| hsa-miR-1 | 7.766 | **hsa-miR-203** | 1.64E+04 |
| hsa-miR-625* | 7.256 | **hsa-miR-886-3p** | 1.60E+04 |
| hsa-miR-603 | 4.716 | **hsa-miR-224** | 1.59E+04 |
| hsa-miR-548d-5p | 4.564 | **hsa-miR-215** | 1.57E+04 |
| hsa-miR-654-3p | 4.332 | **hsa-miR-361** | 1.56E+04 |
| hsa-miR-384 | 4.239 | **hsa-miR-34a** | 1.54E+04 |
| hsa-miR-545* | 4.096 | **hsa-miR-214** | 1.54E+04 |
| hsa-miR-32 | 4.092 | **hsa-miR-486-3p** | 1.40E+04 |
| hsa-miR-145* | 3.981 | **hsa-miR-181a-2*** | 8.58E+03 |
| hsa-miR-99b* | 3.940 | **hsa-miR-543** | 8.58E+03 |
| hsa-miR-516-3p | 3.880 | **hsa-miR-744*** | 8.38E+03 |
| hsa-miR-590-3P | 3.814 | **hsa-miR-199a** | 8.29E+03 |
| hsa-miR-126* | 3.785 | **hsa-miR-183*** | 8.25E+03 |
| hsa-miR-363 | 3.467 | **hsa-miR-365** | 8.09E+03 |
| hsa-miR-627 | 2.248 | **hsa-miR-505** | 7.99E+03 |
| hsa-miR-542-3p | 2.120 | **hsa-miR-212** | 7.86E+03 |
| hsa-miR-422a | 2.081 | **hsa-miR-9** | 7.79E+03 |
| hsa-miR-494 | 2.074 | **hsa-miR-410** | 7.74E+03 |
| hsa-miR-140-3p | 2.068 | **hsa-miR-146b-3p** | 7.73E+03 |
| hsa-miR-155 | 2.041 | **hsa-miR-302c** | 7.31E+03 |
| hsa-miR-885-5p | 2.040 | **hsa-miR-326** | 4.97E+03 |
| hsa-miR-19b-1* | 2.023 | **hsa-miR-106b*** | 4.21E+03 |
|  |  | **hsa-miR-769-5p** | 4.17E+03 |
|  |  | **hsa-miR-1300** | 4.16E+03 |
|  |  | **hsa-miR-1254** | 4.14E+03 |
|  |  | **hsa-miR-181c** | 4.05E+03 |
|  |  | **hsa-miR-629** | 3.93E+03 |
|  |  | **hsa-miR-889** | 3.93E+03 |
|  |  | **hsa-miR-500** | 3.91E+03 |
|  |  | **hsa-miR-29b** | 3.91E+03 |
|  |  | **hsa-miR-30d*** | 2.20E+03 |
|  |  | **hsa-miR-1227** | 2.15E+03 |
|  |  | **hsa-miR-222*** | 2.09E+03 |
|  |  | **hsa-miR-16-1*** | 2.08E+03 |
|  |  | **hsa-miR-15a*** | 2.05E+03 |
|  |  | **hsa-miR-487b** | 2.02E+03 |
|  |  | **hsa-miR-205** | 1.99E+03 |
|  |  | **hsa-miR-452** | 1.96E+03 |
|  |  | **hsa-miR-708** | 1.91E+03 |
|  |  | **hsa-miR-1262** | 1.11E+03 |
|  |  | **hsa-miR-24-2*** | 1.10E+03 |
|  |  | **hsa-miR-214*** | 1.09E+03 |
|  |  | **hsa-miR-628-3p** | 1.09E+03 |
|  |  | **hsa-miR-136*** | 1.09E+03 |
|  |  | **hsa-miR-34b** | 1.09E+03 |
|  |  | **hsa-miR-29a*** | 1.06E+03 |
|  |  | **hsa-miR-26a-1*** | 1.06E+03 |
|  |  | **hsa-miR-27b*** | 1.05E+03 |
|  |  | **hsa-miR-411*** | 1.05E+03 |
|  |  | **hsa-miR-154*** | 1.05E+03 |
|  |  | **hsa-miR-382** | 1.04E+03 |
|  |  | **hsa-miR-362** | 1.03E+03 |
|  |  | **hsa-miR-219** | 1.03E+03 |
|  |  | **hsa-miR-548J** | 1.03E+03 |
|  |  | **hsa-miR-10b** | 1.00E+03 |
|  |  | **hsa-miR-579** | 9.91E+02 |
|  |  | **hsa-miR-671-3p** | 9.87E+02 |
|  |  | **hsa-miR-200b** | 7.89E+02 |
|  |  | **hsa-miR-29b-1*** | 5.75E+02 |
|  |  | **hsa-miR-409-3p** | 5.50E+02 |
|  |  | **hsa-miR-616** | 5.47E+02 |
|  |  | **hsa-let-7f-2*** | 5.43E+02 |
|  |  | **hsa-miR-1271** | 5.42E+02 |
|  |  | **hsa-miR-141** | 5.41E+02 |
|  |  | **hsa-miR-99a*** | 5.33E+02 |
|  |  | **hsa-miR-617** | 5.27E+02 |
|  |  | **hsa-miR-455** | 5.24E+02 |
|  |  | **hsa-miR-34c** | 5.11E+02 |
|  |  | **hsa-miR-449b** | 5.03E+02 |
|  |  | **hsa-miR-337-5p** | 4.83E+02 |
|  |  | **hsa-miR-551b** | 4.81E+02 |
|  |  | **hsa-miR-330-5p** | 4.01E+02 |
|  |  | **hsa-miR-374a*** | 2.77E+02 |
|  |  | **hsa-miR-520c-3p** | 2.71E+02 |
|  |  | **hsa-miR-93*** | 2.68E+02 |
|  |  | **hsa-miR-29b-2*** | 2.56E+02 |
|  |  | **hsa-miR-539** | 2.52E+02 |
|  |  | **hsa-miR-369-3p** | 2.47E+02 |
|  |  | **hsa-miR-338-5P** | 2.40E+02 |
|  |  | **hsa-miR-493** | 2.38E+02 |
|  |  | **hsa-miR-302b** | 1.54E+02 |
|  |  | **hsa-miR-1201** | 1.43E+02 |
|  |  | **hsa-miR-635** | 1.40E+02 |
|  |  | **hsa-miR-26a-2*** | 1.40E+02 |
|  |  | **hsa-miR-520D-3P** | 1.39E+02 |
|  |  | **hsa-miR-1244** | 1.38E+02 |
|  |  | **hsa-miR-92a-1*** | 1.37E+02 |
|  |  | **hsa-miR-10a*** | 1.35E+02 |
|  |  | **hsa-miR-548L** | 1.33E+02 |
|  |  | **hsa-miR-577** | 1.31E+02 |
|  |  | **hsa-miR-591** | 1.29E+02 |
|  |  | **hsa-miR-373** | 1.28E+02 |
|  |  | **hsa-miR-888** | 1.26E+02 |
|  |  | **hsa-miR-193a-3p** | 1.26E+02 |
|  |  | **hsa-miR-411** | 1.25E+02 |
|  |  | **hsa-miR-599** | 1.25E+02 |
|  |  | **hsa-miR-454*** | 1.24E+02 |
|  |  | **hsa-miR-429** | 1.24E+02 |
|  |  | **hsa-miR-193b** | 1.20E+02 |
|  |  | **hsa-miR-380-5p** | 1.15E+02 |
|  |  | **hsa-miR-125a-3p** | 1.13E+02 |
|  |  | **hsa-miR-519a** | 78.24198138 |
|  |  | **hsa-miR-27a*** | 65.64542662 |
|  |  | **hsa-miR-517c** | 62.71888471 |
|  |  | **hsa-miR-127** | 62.40767241 |
|  |  | **hsa-miR-28-3p** | 62.34878754 |
|  |  | **hsa-miR-193a-5p** | 60.66371731 |
|  |  | **hsa-miR-15b** | 59.97573385 |
|  |  | **hsa-miR-340*** | 34.46987113 |
|  |  | **hsa-miR-1291** | 34.20097147 |
|  |  | **hsa-miR-1274B** | 33.60179038 |
|  |  | **hsa-miR-548c-5p** | 31.67087892 |
|  |  | **hsa-miR-502-3p** | 31.58805057 |
|  |  | **hsa-miR-484** | 30.87832456 |
|  |  | **hsa-miR-28** | 30.33663158 |
|  |  | **hsa-miR-30e-3p** | 18.3251741 |
|  |  | **hsa-miR-720** | 17.34903243 |
|  |  | **hsa-miR-30d** | 17.32637809 |
|  |  | **hsa-miR-20a*** | 17.03894375 |
|  |  | **hsa-miR-324-3p** | 15.83994038 |
|  |  | **hsa-miR-21** | 15.82191586 |
|  |  | **hsa-miR-92a** | 15.66715403 |
|  |  | **hsa-miR-331** | 15.56085039 |
|  |  | **hsa-miR-125a-5p** | 15.53542172 |
|  |  | **hsa-miR-320** | 15.46925915 |
|  |  | **hsa-miR-30b** | 15.41437939 |
|  |  | **hsa-miR-99b** | 15.37004304 |
|  |  | **hsa-miR-744** | 15.23397998 |
|  |  | **hsa-miR-148a** | 15.11057338 |
|  |  | **hsa-miR-30c** | 14.93474231 |
|  |  | **hsa-miR-423-5p** | 14.54399799 |
|  |  | **hsa-miR-376c** | 14.1346769 |
|  |  | **hsa-miR-148b** | 12.934753 |
|  |  | **hsa-miR-330** | 9.751667935 |
|  |  | **hsa-miR-130b** | 8.968694506 |
|  |  | **hsa-miR-10a** | 8.954153187 |
|  |  | **hsa-miR-511** | 8.506997322 |
|  |  | **hsa-miR-320B** | 8.492951433 |
|  |  | **hsa-miR-151-5P** | 8.356811534 |
|  |  | **hsa-miR-186** | 8.289419071 |
|  |  | **hsa-miR-10b*** | 8.284417723 |
|  |  | **hsa-miR-766** | 8.273173283 |
|  |  | **hsa-miR-151-3p** | 8.160210114 |
|  |  | **hsa-miR-181a** | 8.029298786 |
|  |  | **hsa-let-7d** | 7.859293885 |
|  |  | **hsa-miR-339-3p** | 7.851574549 |
|  |  | **hsa-miR-199a-3p** | 7.842446053 |
|  |  | **hsa-miR-345** | 7.81766086 |
|  |  | **hsa-miR-150** | 7.806046301 |
|  |  | **hsa-miR-142-3p** | 7.803807009 |
|  |  | **hsa-miR-106b** | 7.792527818 |
|  |  | **hsa-miR-27b** | 7.764965959 |
|  |  | **hsa-miR-139-5p** | 7.742333216 |
|  |  | **hsa-miR-29c** | 7.713207913 |
|  |  | **hsa-miR-135a** | 7.707403921 |
|  |  | **hsa-miR-381** | 7.688062156 |
|  |  | **hsa-miR-27a** | 7.657433856 |
|  |  | **hsa-miR-324-5p** | 7.655353509 |
|  |  | **hsa-miR-25** | 7.650886389 |
|  |  | **hsa-miR-223** | 7.643218638 |
|  |  | **hsa-miR-222** | 7.638302202 |
|  |  | **hsa-miR-24** | 7.620833364 |
|  |  | **hsa-miR-328** | 7.597384304 |
|  |  | **hsa-miR-152** | 7.59603946 |
|  |  | **hsa-miR-125b** | 7.570032531 |
|  |  | **hsa-miR-376a** | 7.548526225 |
|  |  | **hsa-miR-126** | 7.495322946 |
|  |  | **hsa-miR-628-5p** | 5.270986511 |
|  |  | **hsa-miR-296** | 4.293154224 |
|  |  | **hsa-miR-18a*** | 4.219093325 |
|  |  | **hsa-miR-1274A** | 4.197272993 |
|  |  | **hsa-miR-223*** | 4.083364061 |
|  |  | **hsa-miR-17** | 4.023080762 |
|  |  | **hsa-miR-184** | 4.013711718 |
|  |  | **hsa-miR-26b*** | 4.012877177 |
|  |  | **hsa-miR-598** | 3.989638417 |
|  |  | **hsa-miR-146b** | 3.953936221 |
|  |  | **hsa-miR-133a** | 3.951318936 |
|  |  | **hsa-miR-128a** | 3.938698539 |
|  |  | **hsa-miR-20a** | 3.898520984 |
|  |  | **hsa-miR-342-3p** | 3.874366947 |
|  |  | **hsa-miR-26a** | 3.870311807 |
|  |  | **hsa-miR-18a** | 3.855550862 |
|  |  | **hsa-miR-642** | 3.845686076 |
|  |  | **hsa-miR-483-5p** | 3.84505091 |
|  |  | **hsa-miR-145** | 3.842590664 |
|  |  | **hsa-miR-221** | 3.800842385 |
|  |  | **hsa-miR-335** | 3.790771509 |
|  |  | **hsa-miR-574-3p** | 3.782625634 |
|  |  | **hsa-miR-106a** | 3.780720505 |
|  |  | **hsa-miR-146a** | 3.77249686 |
|  |  | **hsa-miR-532-3p** | 3.767328924 |
|  |  | **hsa-miR-218** | 3.754177856 |
|  |  | **hsa-miR-101** | 3.74731953 |
|  |  | **hsa-let-7g** | 3.700219872 |
|  |  | **hsa-miR-185** | 3.575928524 |
|  |  | **hsa-miR-301** | 3.561430903 |
|  |  | **hsa-let-7e** | 3.554252065 |
|  |  | **hsa-miR-572** | 2.333162837 |
|  |  | **hsa-miR-629** | 2.114134261 |
|  |  | **hsa-miR-9*** | 2.089940689 |
|  |  | **hsa-miR-645** | 2.084724873 |
|  |  | **hsa-miR-545** | 2.077454673 |
|  |  | **hsa-miR-505*** | 2.076318119 |
|  |  | **hsa-miR-204** | 2.00715913 |
|  |  |  |  |
