# Supplementary material for: Differentially expressed microRNAs in the serum of cervical squamous cell carcinoma patients before and after surgery
Source: J Hematol Oncol. 2014 Jan 10;7:6. doi: 10.1186/1756-8722-7-6 (PMC3892020; doi:10.1186/1756-8722-7-6)
Supplement: Additional file 2: Table S2 — MiRNAs fluctuated more than ten folds between cervical squamous cell carcinoma serum samples and negative controls. [file 1756-8722-7-6-S2.docx]

**Table S2. MiRNAs fluctuated more than ten folds between cervical squamous cell carcinoma serum samples and negative controls**

| Downregulated microRNA | Fold Change | Upregulated microRNA | Fold Change |
| --- | --- | --- | --- |
| hsa-miR-508 | 3.894E+04 | **hsa-miR-646** | 3.040E+07 |
| hsa-miR-1825 | 1.606E+04 | **hsa-miR-1183** | 1.826E+07 |
| hsa-miR-202 | 1.061E+04 | **hsa-miR-1233** | 1.147E+06 |
| hsa-miR-139-3p | 9.467E+03 | **hsa-miR-661** | 1.076E+06 |
| hsa-miR-523 | 8.487E+03 | **hsa-miR-486** | 5.239E+05 |
| hsa-miR-618 | 2.307E+03 | **hsa-miR-122** | 5.016E+05 |
| hsa-miR-103 | 2.141E+03 | **hsa-miR-130a** | 1.282E+05 |
| hsa-miR-141* | 2.023E+03 | **hsa-miR-15a** | 1.094E+05 |
| hsa-miR-875-5p | 5.322E+02 | **hsa-miR-143** | 3.274E+04 |
| hsa-miR-1243 | 2.556E+02 | **hsa-miR-31** | 3.164E+04 |
| hsa-miR-335* | 2.461E+02 | **hsa-miR-532** | 3.152E+04 |
| hsa-miR-601 | 2.434E+02 | **hsa-miR-664** | 3.145E+04 |
| hsa-miR-519b-3p | 2.428E+02 | **hsa-miR-372** | 3.110E+04 |
| hsa-miR-548d | 1.416E+02 | **hsa-miR-370** | 3.080E+04 |
| hsa-miR-548c | 1.341E+02 | **hsa-miR-200c** | 3.010E+04 |
| hsa-miR-190b | 1.222E+02 | **hsa-miR-378** | 1.735E+04 |
| hsa-miR-489 | 72.113 | **hsa-miR-133b** | 1.699E+04 |
| hsa-miR-206 | 61.472 | **hsa-miR-132** | 1.641E+04 |
| hsa-miR-548a | 33.213 | **hsa-miR-203** | 1.637E+04 |
| hsa-miR-191* | 30.756 | **hsa-miR-215** | 1.566E+04 |
| hsa-miR-515-3p | 17.646 | **hsa-miR-214** | 1.539E+04 |
| hsa-miR-195 | 16.435 | **hsa-miR-199a** | 8.293E+03 |
| hsa-miR-302a | 16.270 | **hsa-miR-365** | 8.092E+03 |
| hsa-miR-485-3p | 15.448 | **hsa-miR-505** | 7.995E+03 |
| hsa-miR-192* | 15.408 | **hsa-miR-212** | 7.861E+03 |
| hsa-miR-758 | 9.898 | **hsa-miR-9** | 7.788E+03 |
|  |  | **hsa-miR-181c** | 4.054E+03 |
|  |  | **hsa-miR-29b** | 3.913E+03 |
|  |  | **hsa-miR-205** | 1.986E+03 |
|  |  | **hsa-miR-452** | 1.964E+03 |
|  |  | **hsa-miR-708** | 1.908E+03 |
|  |  | **hsa-miR-200b** | 7.889E+02 |
|  |  | **hsa-miR-1271** | 5.419E+02 |
|  |  | **hsa-miR-141** | 5.411E+02 |
|  |  | **hsa-miR-551b** | 4.808E+02 |
|  |  | **hsa-miR-493** | 2.385E+02 |
|  |  | **hsa-miR-302b** | 1.541E+02 |
|  |  | **hsa-miR-1244** | 1.376E+02 |
|  |  | **hsa-miR-21** | 15.822 |
|  |  | **hsa-miR-92a** | 15.667 |
