# Supplementary material for: Differentially expressed microRNAs in the serum of cervical squamous cell carcinoma patients before and after surgery
Source: J Hematol Oncol. 2014 Jan 10;7:6. doi: 10.1186/1756-8722-7-6 (PMC3892020; doi:10.1186/1756-8722-7-6)
Supplement: Additional file 3: Table S3 — The global miRNA species showed relative expression profiles between cervical squamous cell carcinoma serum samples before and after surgery. [file 1756-8722-7-6-S3.docx]

**Table S3.** **The global miRNA species showed relative expression profiles between cervical squamous cell carcinoma serum samples before and after surgery**

| Global Upregulated microRNA | Fold Change | Global Downregulated microRNA | Fold Change |
| --- | --- | --- | --- |
| hsa-miR-1243 | 2.300E+10 | **hsa-miR-646** | 9.692E+04 |
| hsa-miR-198 | 8.598E+06 | **hsa-miR-1233** | 7.199E+03 |
| hsa-miR-216a | 2.105E+06 | **hsa-miR-15a** | 2.658E+03 |
| hsa-miR-188-3p | 1.377E+06 | **hsa-miR-664** | 2.027E+03 |
| hsa-miR-518f | 6.025E+05 | **hsa-miR-370** | 1.013E+03 |
| hsa-miR-208b | 3.174E+05 | **hsa-miR-214** | 2.667E+02 |
| hsa-miR-369-5p | 1.624E+05 | **hsa-miR-181c** | 2.620E+02 |
| hsa-let-7f | 1.319E+05 | **hsa-miR-372** | 1.819E+02 |
| hsa-miR-216b | 6.720E+04 | **hsa-miR-1227** | 9.692E+04 |
| hsa-miR-219-1-3p | 3.130E+04 | **hsa-miR-24-2*** | 92.608 |
| hsa-miR-130a* | 6.487E+03 | **hsa-miR-34b** | 92.010 |
| hsa-miR-16-2* | 6.372E+03 | **hsa-miR-27b*** | 88.987 |
| hsa-miR-154 | 4.341E+03 | **hsa-miR-411*** | 88.502 |
| hsa-miR-302a | 4.164E+03 | **hsa-miR-548J** | 86.706 |
| hsa-miR-636 | 4.027E+03 | **hsa-miR-132** | 66.364 |
| hsa-miR-492 | 2.097E+03 | **hsa-miR-323-3p** | 59.715 |
| hsa-miR-624 | 1.934E+03 | **hsa-miR-200b** | 51.915 |
| hsa-miR-523 | 1.920E+03 | **hsa-miR-29b-1*** | 48.601 |
| hsa-miR-367 | 1.058E+03 | **hsa-let-7f-2*** | 45.852 |
| hsa-miR-618 | 1.037E+03 | **hsa-miR-1271** | 45.801 |
| hsa-miR-502 | 9.991E+02 | **hsa-miR-617** | 44.496 |
| hsa-miR-218-1* | 6.932E+02 | **hsa-miR-1254** | 43.979 |
| hsa-miR-1265 | 3.768E+02 | **hsa-miR-339-5p** | 35.174 |
| hsa-miR-624 | 3.686E+02 | **hsa-miR-449b** | 33.097 |
| hsa-miR-1276 | 3.682E+02 | **hsa-miR-212** | 31.846 |
| hsa-miR-708 | 2.832E+02 | **hsa-miR-744*** | 22.644 |
| hsa-miR-627 | 2.714E+02 | **hsa-miR-106b*** | 22.309 |
| hsa-miR-520f | 2.502E+02 | **hsa-miR-29b-2*** | 21.609 |
| hsa-miR-338-3p | 2.469E+02 | **hsa-miR-485-3p** | 16.990 |
| hsa-miR-105 | 2.412E+02 | **hsa-miR-200c** | 15.731 |
| hsa-miR-132* | 2.222E+02 | **hsa-miR-493** | 15.692 |
| hsa-miR-135b* | 1.893E+02 | **hsa-miR-489** | 15.346 |
| hsa-miR-213 | 1.832E+02 | **hsa-miR-1201** | 12.054 |
| hsa-miR-497 | 1.657E+02 | **hsa-miR-635** | 11.852 |
| hsa-miR-542-3p | 1.333E+02 | **hsa-miR-29a*** | 11.497 |
| hsa-miR-191* | 94.037 | **hsa-miR-10a*** | 11.387 |
| hsa-miR-330-5p | 88.143 | **hsa-miR-548L** | 11.237 |
| hsa-miR-219 | 64.360 | **hsa-miR-577** | 11.112 |
| hsa-miR-509-5p | 63.838 | **hsa-miR-335*** | 11.022 |
| hsa-miR-382 | 59.530 | **hsa-miR-26b*** | 10.614 |
| hsa-miR-411 | 58.101 | **hsa-miR-599** | 10.563 |
| hsa-miR-192* | 45.437 | **hsa-miR-875-5p** | 10.331 |
| hsa-miR-302b | 35.606 | **hsa-miR-296** | 8.503 |
| hsa-miR-331-5p | 31.849 | **hsa-miR-373** | 8.444 |
| hsa-miR-548a-5p | 27.560 | **hsa-miR-645** | 8.322 |
| hsa-miR-31* | 23.518 | **hsa-miR-888** | 8.297 |
| hsa-miR-581 | 21.881 | **hsa-miR-429** | 8.172 |
| hsa-miR-550 | 21.763 | **hsa-miR-339-3p** | 8.032 |
| hsa-miR-572 | 19.954 | **hsa-miR-505** | 7.751 |
| hsa-miR-517b | 15.872 | **hsa-miR-505*** | 5.959 |
| hsa-miR-944 | 11.750 | **hsa-miR-942** | 5.747 |
| hsa-miR-548P | 11.538 | **hsa-miR-661** | 5.693 |
| hsa-miR-122* | 9.925 | **hsa-miR-16-1*** | 5.391 |
| hsa-miR-376a* | 9.803 | **hsa-miR-519a** | 5.149 |
| hsa-miR-301b | 8.520 | **hsa-miR-517c** | 4.127 |
| hsa-miR-551b | 8.453 | **hsa-miR-365** | 4.026 |
| hsa-miR-138 | 8.406 | **hsa-miR-193a-5p** | 4.006 |
| hsa-miR-362-3p | 8.222 | **hsa-miR-548c-5p** | 3.967 |
| hsa-miR-194 | 8.072 | **hsa-miR-889** | 3.918 |
| hsa-miR-483-5p | 7.971 | **hsa-miR-193b** | 3.915 |
| hsa-miR-101 | 7.964 | **hsa-miR-342-3p** | 3.866 |
| hsa-miR-10b | 7.729 | **hsa-miR-302c** | 3.593 |
| hsa-miR-1244 | 5.689 | **hsa-miR-148b** | 3.427 |
| hsa-miR-148b* | 5.594 | **hsa-miR-1262** | 2.887 |
| hsa-miR-769-5p | 5.159 | **hsa-miR-19b-1*** | 2.868 |
| hsa-miR-202 | 4.819 | **hsa-miR-214*** | 2.849 |
| hsa-miR-146b-3p | 4.545 | **hsa-miR-30a-3p** | 2.806 |
| hsa-miR-329 | 4.326 | **hsa-miR-93*** | 2.796 |
| hsa-miR-324-5p | 4.239 | **hsa-miR-425*** | 2.770 |
| hsa-miR-135a | 4.131 | **hsa-miR-18a*** | 2.701 |
| hsa-miR-374 | 4.103 | **hsa-miR-151-3p** | 2.699 |
| hsa-miR-130b | 4.084 | **hsa-miR-1300** | 2.673 |
| hsa-miR-579 | 4.074 | **hsa-miR-20a*** | 2.669 |
| hsa-miR-190 | 4.014 | **hsa-miR-183*** | 2.596 |
| hsa-miR-181a | 4.013 | **hsa-miR-545** | 2.256 |
| hsa-miR-19a | 3.992 | **hsa-miR-204** | 2.088 |
| hsa-miR-29b | 3.986 | **hsa-miR-196b** | 2.054 |
| hsa-miR-195 | 3.979 | **hsa-miR-128a** | 2.022 |
| hsa-miR-422a | 3.969 | **hsa-miR-185** | 2.008 |
| hsa-miR-18a | 3.963 |  |  |
| hsa-miR-340 | 3.959 |  |  |
| hsa-miR-381 | 3.954 |  |  |
| hsa-miR-597 | 3.950 |  |  |
| hsa-miR-140-3p | 3.946 |  |  |
| hsa-miR-9 | 3.942 |  |  |
| hsa-miR-34a | 3.923 |  |  |
| hsa-miR-95 | 3.909 |  |  |
| hsa-miR-142-3p | 3.883 |  |  |
| hsa-miR-29a | 3.880 |  |  |
| hsa-miR-34c | 3.775 |  |  |
| hsa-miR-142-5p | 3.773 |  |  |
| hsa-miR-362 | 3.734 |  |  |
| hsa-miR-548d-5p | 3.696 |  |  |
| hsa-miR-1255B | 3.028 |  |  |
| hsa-miR-99b* | 2.937 |  |  |
| hsa-miR-320B | 2.913 |  |  |
| hsa-miR-144* | 2.912 |  |  |
| hsa-miR-591 | 2.858 |  |  |
| hsa-miR-1274A | 2.830 |  |  |
| hsa-miR-720 | 2.805 |  |  |
| hsa-miR-409-3p | 2.790 |  |  |
| hsa-miR-206 | 2.787 |  |  |
| hsa-miR-190b | 2.745 |  |  |
| hsa-miR-136* | 2.665 |  |  |
| hsa-miR-628-5p | 2.562 |  |  |
| hsa-miR-663B | 2.542 |  |  |
| hsa-miR-99a | 2.283 |  |  |
| hsa-miR-486-3p | 2.239 |  |  |
| hsa-miR-532 | 2.186 |  |  |
| hsa-miR-410 | 2.149 |  |  |
| hsa-miR-598 | 2.112 |  |  |
| hsa-miR-125a-3p | 2.111 |  |  |
| hsa-miR-758 | 2.104 |  |  |
| hsa-let-7a | 2.085 |  |  |
| hsa-miR-363 | 2.079 |  |  |
| hsa-miR-548b-5p | 2.059 |  |  |
| hsa-miR-548a | 2.053 |  |  |
| hsa-miR-494 | 2.048 |  |  |
| hsa-miR-126 | 2.048 |  |  |
| hsa-miR-25 | 2.043 |  |  |
| hsa-miR-361 | 2.034 |  |  |
| hsa-miR-337-5p | 2.030 |  |  |
| hsa-miR-26a | 2.027 |  |  |
| hsa-miR-486 | 2.025 |  |  |
| hsa-miR-28-3p | 2.021 |  |  |
| hsa-miR-92a | 2.016 |  |  |
| hsa-miR-331 | 2.015 |  |  |
| hsa-miR-106b | 2.012 |  |  |
| hsa-miR-184 | 2.010 |  |  |
| hsa-miR-197 | 2.008 |  |  |
| hsa-miR-125b | 2.001 |  |  |
