# Supplementary material for: Differentially expressed microRNAs in the serum of cervical squamous cell carcinoma patients before and after surgery
Source: J Hematol Oncol. 2014 Jan 10;7:6. doi: 10.1186/1756-8722-7-6 (PMC3892020; doi:10.1186/1756-8722-7-6)
Supplement: Additional file 4: Table S4 — MiRNAs fluctuated more than ten folds between cervical squamous cell carcinoma serum samples before and after surgery. [file 1756-8722-7-6-S4.docx]

**Table S4. MiRNAs fluctuated more than ten folds between cervical squamous cell carcinoma serum samples before and after surgery**

| Upregulated microRNA | Fold Change | Downregulated microRNA | Fold Change |
| --- | --- | --- | --- |
| hsa-miR-1243 | 2.300E+10 | **hsa-miR-646** | 2.569E+06 |
| hsa-miR-198 | 8.598E+06 | **hsa-miR-1233** | 9.692E+04 |
| hsa-miR-216a | 2.105E+06 | **hsa-miR-15a** | 7.199E+03 |
| hsa-miR-188-3p | 1.377E+06 | **hsa-miR-664** | 2.658E+03 |
| hsa-miR-518f | 6.025E+05 | **hsa-miR-370** | 2.027E+03 |
| hsa-miR-208b | 3.174E+05 | **hsa-miR-214** | 1.013E+03 |
| hsa-miR-369-5p | 1.624E+05 | **hsa-miR-181c** | 2.667E+02 |
| hsa-let-7f | 1.319E+05 | **hsa-miR-372** | 2.620E+02 |
| hsa-miR-216b | 6.720E+04 | **hsa-miR-1227** | 1.819E+02 |
| hsa-miR-154 | 4.341E+03 | **hsa-miR-34b** | 92.00956873 |
| hsa-miR-302a | 4.164E+03 | **hsa-miR-548J** | 86.70630499 |
| hsa-miR-636 | 4.027E+03 | **hsa-miR-132** | 66.36372675 |
| hsa-miR-492 | 2.097E+03 | **hsa-miR-323-3p** | 59.71460815 |
| hsa-miR-624 | 1.934E+03 | **hsa-miR-200b** | 51.91534052 |
| hsa-miR-523 | 1.920E+03 | **hsa-miR-1271** | 45.80089864 |
| hsa-miR-367 | 1.058E+03 | **hsa-miR-617** | 44.49591535 |
| hsa-miR-618 | 1.037E+03 | **hsa-miR-1254** | 43.97891174 |
| hsa-miR-502 | 9.991E+02 | **hsa-miR-339-5p** | 35.17398774 |
| hsa-miR-218-1* | 6.932E+02 | **hsa-miR-449b** | 33.09749213 |
| hsa-miR-708 | 2.832E+02 | **hsa-miR-212** | 31.84568501 |
| hsa-miR-627 | 2.714E+02 | **hsa-miR-744*** | 22.64444068 |
| hsa-miR-105 | 2.412E+02 | **hsa-miR-106b*** | 22.30906276 |
| hsa-miR-213 | 1.832E+02 | **hsa-miR-485-3p** | 16.99003595 |
| hsa-miR-497 | 1.657E+02 | **hsa-miR-200c** | 15.73123467 |
| hsa-miR-542-3p | 1.333E+02 | **hsa-miR-493** | 15.69220301 |
| hsa-miR-330-5p | 88.143 | **hsa-miR-489** | 15.34637491 |
| hsa-miR-219 | 64.360 | **hsa-miR-1201** | 12.05353993 |
| hsa-miR-509-5p | 63.838 | **hsa-miR-635** | 11.85165804 |
| hsa-miR-382 | 59.530 | **hsa-miR-548L** | 11.23711669 |
| hsa-miR-411 | 58.101 | **hsa-miR-577** | 11.11221875 |
| hsa-miR-302b | 35.606 | **hsa-miR-599** | 10.56341934 |
| hsa-miR-331-5p | 31.849 | **hsa-miR-875-5p** | 10.33083528 |
| hsa-miR-581 | 21.881 |  |  |
| hsa-miR-550 | 21.763 |  |  |
| hsa-miR-572 | 19.954 |  |  |
| hsa-miR-517b | 15.872 |  |  |
| hsa-miR-944 | 11.750 |  |  |
